# A Large Nonmetastatic Anaplastic Thyroid Cancer with Complete Thyroidal Confinement

**DOI:** 10.1155/2011/583978

**Published:** 2011-09-07

**Authors:** Jeffrey C. Xing, Justin A. Bishop, Nestoras Mathioudakis, Nishant Agrawal, Ralph P. Tufano

**Affiliations:** ^1^Department of Otolaryngology-Head and Neck Surgery, Johns Hopkins University School of Medicine, Baltimore, MD 21287-0910, USA; ^2^Department of Pathology, Johns Hopkins University School of Medicine, Baltimore, MD 21287-0910, USA; ^3^Department of Medicine, Johns Hopkins University School of Medicine, Baltimore, MD 21287-0910, USA

## Abstract

Anaplastic thyroid cancer (ATC) is rare but extremely aggressive, which accounts for about 2% of all thyroid cancers yet nearly 50% of thyroid-cancer-associated deaths in the United States. The median survival time from diagnosis is 5 months, with a 1-year survival rate of only 20%. We report here a case of ATC in a 56-year-old man who survived a large ATC. Preoperative fine-needle aspiration biopsy study to a large right thyroid mass suggested ATC. Total thyroidectomy with radical lateral neck and central neck dissection removed a well-circumscribed 9.5 cm tumor without extrathyroidal extension or lymphovascular invasion. All 73 lymph nodes removed were negative for metastasis. The tumor consisted of highly pleomorphic, undifferentiated cells with large zones of necrosis and loss of thyroid transcription factor-1 and thyroglobulin expression. A focal well-differentiated component and PAX8 expression confirmed its thyroid follicular cell origin. Nine months after postsurgical adjuvant concurrent radiation therapy and chemotherapy, the patient remained well without clinical, biochemical, and radiographical evidence for cancer recurrence. This is an unusual case of ATC in that it is one of the largest ATC tumors reported to display mild pathologic behavior and relatively long-term patient survival.

## 1. Case Report

**Figure 1:**
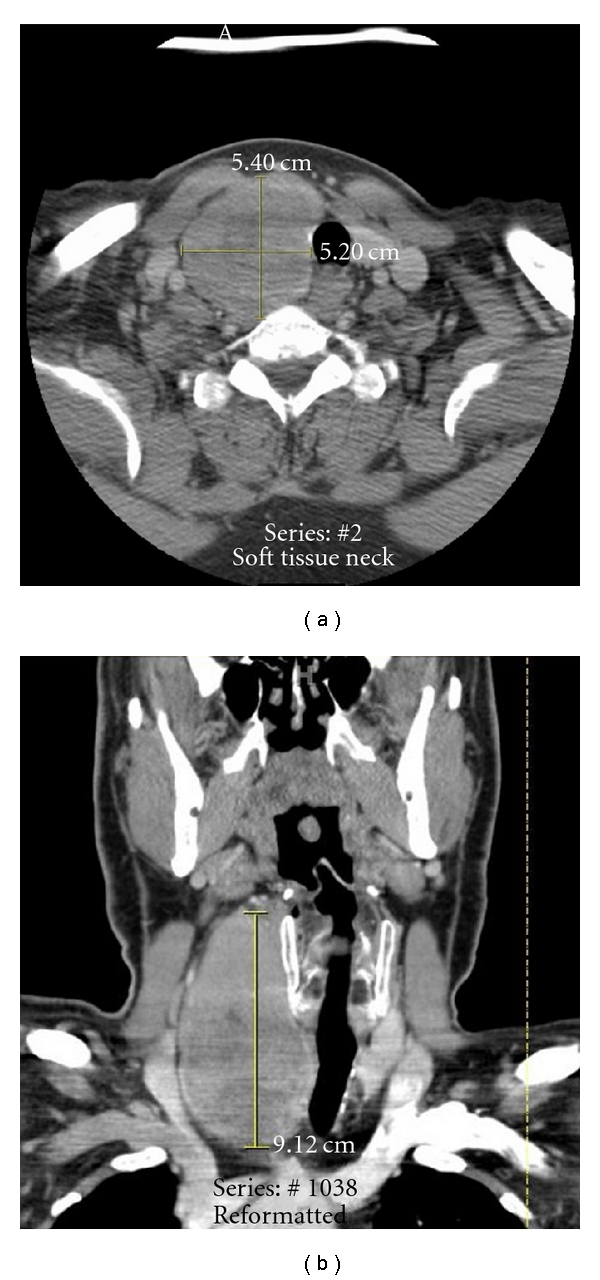
Preoperative computed tomography (CT) of the anaplastic thyroid carcinoma. (a) Axial view with contrast revealing a heterogeneous mass that is intimately associated with the trachea and pushes it toward the left side of the neck but free of the common carotid artery and the esophagus. (b) Coronal view with contrast demonstrating the craniocaudal extent and intimate tracheal involvement of the tumor.

The patient is a 56-year-old white man who presented to his primary care physician one day after he noticed a mass in his right neck. Computed tomography (CT) scan confirmed a large mass in his right neck arising from the right thyroid lobe, measuring 5.4 × 5.2 × 9.1 cm (Figure 1). The CT also revealed a few lymph nodes without clear fatty hila which measured 1-2 cm in the right neck and upper mediastinum. Two weeks later, the patient underwent a fine-needle aspiration biopsy of the thyroid mass, which revealed highly malignant cells in a background of blood and necrotic debris. The patient was then referred to our institution for further management of suspected ATC. 

The patient's history was notable for a 55-pound weight loss and fatigue, but he did not have compressive symptoms, such as dysphagia or dyspnea, due to the mass. There was no antecedent history of radiation exposure to the head and neck. Physical exam revealed a large, visible mass extending from the thyroid bed to the lateral aspect of the right neck, occupying virtually the entire right anterior and lateral neck. It extended also toward the left side beyond the midline. This large mass was firm and nontender upon palpation and was relatively immobile. The left thyroid lobe was unremarkable. The trachea was difficult to examine as it was covered by the mass anteriorly. Neck ultrasonography revealed a large, hypoechoic, irregular-shaped mass containing punctate calcifications. A whole-body fluorodeoxyglucose (^18^F) positron emission tomography (PET) scan with high-resolution CT demonstrated the right neck mass to be hypermetabolic and revealed also a few hypermetabolic lymph nodes in the right neck and upper mediastinum with no distant metastasis. A Tc-99 MDP whole-body scan showed no metastatic disease in the skeletal system. The esophageal muscularis, trachea, and carotid artery appeared to be well preserved structurally on these imaging studies except for leftward deviation of the trachea. There were no abnormal imaging findings to suggest distant metastasis.

Evaluation by the otolaryngology-head and neck surgery consultation service at our institution, including a fiberoptic laryngoscopy, was unremarkable except for the confirmation of a large right thyroid mass. Based on the clinical and diagnostic data, as well as confirmation of the diagnosis of ATC by our pathology department, our multidisciplinary thyroid tumor team decided to pursue total thyroidectomy with right and central neck dissections. Surgery was performed three days after he transferred to our institution, which was approximately one month from the time the patient first noted the neck mass. A well-circumscribed large thyroid tumor was successfully removed, along with 73 lymph nodes from the central and right lateral compartments of the neck and the upper mediastinum. 

**Figure 2:**
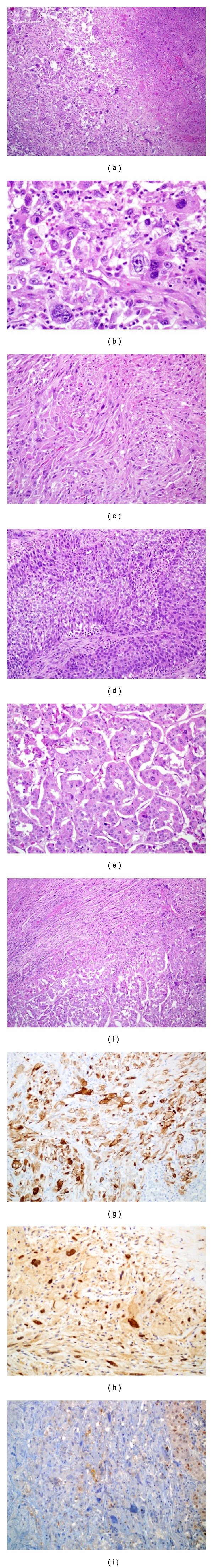
Postoperative histopathology of the resected tumor. (a) The thyroid tumor featured large zones of necrosis (right) (hematoxylin and eosin, ×100). (b) The tumor was composed of highly pleomorphic cells with large, bizarre nuclei containing prominent nucleoli and mitotic figures. Much of the tumor was infiltrated by neutrophils, a common feature of anaplastic thyroid carcinoma (hematoxylin and eosin, ×400). While the predominant pattern of the tumor was pleomorphic/giant cell, there were areas that were spindled (c) hematoxylin and eosin, ×200) or epidermoid (d) hematoxylin and eosin, ×200). (e) Focally admixed with the undifferentiated tumor was a well-differentiated oncocytic component (hematoxylin and eosin, ×200). (f) The well-differentiated component transitioned directly into the predominant anaplastic component. The anaplastic carcinoma was positive for cytokeratins AE1/AE3 (g) (AE1/AE3 immunohistochemistry, ×200) and PAX8 (h) (PAX8 immunohistochemistry, ×200). (i) The well-differentiated component of the tumor (right) expressed thyroid transcription factor-1, while the anaplastic component (left) did not (thyroid transcription factor-1 immunohistochemistry, ×200).

Gross pathologic evaluation of the thyroid revealed one 9.5 cm tumor with central necrosis and hemorrhage. The tumor was partially encapsulated and completely confined within the thyroid gland. Histological evaluation revealed an infiltrative malignant neoplasm with zones of geographic tumor necrosis (Figure 2(a)). The tumor cells were undifferentiated at the light microscopic level, with large and highly pleomorphic nuclei containing prominent nucleoli and numerous atypical mitoses, consistent with giant-cell variant of ATC (Figure 2(b)). Although the neoplasm had a predominantly giant cell pattern, there were also areas of spindled (Figure 2(c)) and epidermoid (Figure 2(d)) histology. These were not osteoclast-like giant cells as seen in a previously reported case of ATC with long survival of the patient [1]. Focally admixed with the undifferentiated tumor was also a well-differentiated oncocytic component (Figure 2(e)), which was localized centrally in the middle of the tumor, accounting for about 10% of the entire tumor. Anaplastic cells were throughout the rest of the tumor. The well-differentiated component transitioned directly into the predominant anaplastic component (Figure 2(f)). The anaplastic carcinoma was positive for cytokeratins AE1/AE3 (Figure 2(g)) and PAX8 (Figure 2(h)). Only the well-differentiated component but not the anaplastic component of the tumor expressed thyroid transcription factor-1 (Figure 2(i)). The well-differentiated component but not the anaplastic component also expressed thyroglobulin (data not shown). These findings were consistent with anaplastic thyroid carcinoma. Vascular invasion was not identified, and the carcinoma did not extend beyond the thyroid. All 73 lymph nodes of the neck dissection were negative for carcinoma.

Thyroxine replacement therapy was started postoperatively. Although there was no evidence to suggest local or distant tumor invasion or metastasis, given the classical aggressive nature of ATC in general, postoperatively the patient received concurrent chemotherapy and external beam radiation with weekly doxorubicin and docetaxel and a total radiation of 6000 cGy over a course of seven weeks. During this treatment course, the patient suffered from mild toxicity with discomfort in his upper chest, tightness in the neck, some dysphagia, anemia, and leukopenia. Nine months postoperatively, the patient remained well and enjoyed his normal life without major issues except for some mild dysphagia, neck tightness, and chest changes from the radiation therapy. During the posttreatment course, the patient had several surveillance imaging studies, including neck ultrasonography, head/neck/chest CT, and whole-body PET/CT scans, which were all negative for cancer recurrence or metastasis. The hypermetabolic lymph nodes on the initial preoperative PET scan were no longer seen on the recent postoperative surveillance PET scan. An echocardiogram was also normal. His serum thyroglobulin and carcinoembryonic antigen (CEA) were all undetectable during this course. 

## 2. Discussion

ATC is a rare but extremely aggressive and rapidly lethal thyroid cancer derived from follicular epithelial thyroid cells; in fact, it is one of the most deadly human cancers [2, 3]. Although this cancer accounts for only about 2% of all thyroid malignancies, it is responsible for nearly half of thyroid-cancer-related human deaths in the United States [4]. An overall analysis of 1771 published cases of ATC patients revealed median patient survival duration of only five months and a median one-year survival rate of 20% [3]. Extensive local invasion and lymph node metastasis of the tumor with distant metastasis to lungs, bones, and other parts of the body are commonly seen at the diagnosis of ATC, leaving the disease incurable and deadly at the early stage after the diagnosis. 

Our case of ATC was unusual in that although the tumor represents one of the largest reported to date, there was no local tumor invasion and even no extrathyroidal, venous, and lymphatic invasion. Extensive neck dissection removed a large number of lymph nodes, but none of them was found to have metastasis. There was also no evidence of distant metastasis either clinically or radiographically. Nine months postoperatively, there was no clinical, biochemical, and imaging evidence to suggest cancer recurrence and the patient lives an essentially normal life, being able to return to his work as a restaurant manager with normal function. Despite the limited followup time interval of nine months, the partially encapsulated nature and complete thyroidal confinement of this large tumor and absence of metastasis at the time of diagnosis predict a more favorable long-term outcome. As such, this case is outstanding and deviates from the normal disease course in classical ATC patients. There have been several case reports on patients with ATC that showed an indolent clinical course with long-term patient survival [5–13]. Most of these studies reported ATC tumors of 2–5 cm with recorded patient survival time of 2-3 years, with the longest survival of more than 10 years in a patient with a 5 cm ATC [11]. The 9.5 cm tumor of our case thus represents one of the largest among the reported ATC tumors while exhibiting no metastatic behavior. As even small ATC tumors are usually lethally invasive and aggressive, the lack of the classical aggressive features in our case and those published similar cases suggest that a subset of ATC tumors may behave differently than classical ATC. Patients in this group of ATC seem to have an excellent prognosis if complete surgical resection can be accomplished. A striking pathological feature of our case was its lack of extrathyroidal invasion and metastasis even though the tumor size was extremely large, reflecting a unique relatively indolent pattern of biological behavior of ATC tumors in this group. This is likely the basis for the good clinical outcome of our case as well as the similar long-term survival cases published. Such cases attest to the need for more fundamental prognostic markers in evaluating and treating ATC patients. ATC pathogenesis stems from a complex array of genetic derangements, including gene mutations and amplifications [2, 3, 14, 15]. We speculate that the genetic alterations (or lack thereof) underlying our case contribute to its unique outcome. It would be interesting and important to identify such genetic alterations that can help understand the biological basis of this subset of ATC and guide their clinical prognostication and management. 

Given the lack of effective medical treatment for ATC currently, aggressive multimodal treatment customized on a case-by-case basis, particularly after early diagnosis, remains the treatment of choice for this cancer [2, 3, 16, 17]. When this patient was seen by the medical endocrine team at our institution, he was immediately referred and arranged for surgical, medical oncology, and radiation oncology consults. Because of the encouraging resectability of this particular tumor shown on the preoperative CT study, our multidisciplinary thyroid tumor review board opted for surgery followed by adjuvant radiation and chemotherapy. Although this case of ATC was not as invasive and aggressive as is usually the case of ATC, the rapid multidisciplinary management and forceful multimodal treatment of this patient were appropriate given the currently limited understanding of ATC. We suggest that, as for classical aggressive ATC, the same approach should remain the management strategy for this unique subset of ATC until we can better understand its biology and reliably predict its clinical outcomes.
